# Supplementary material for: Scoring alignments by embedding vector similarity
Source: Brief Bioinform. 2024 May 1;25(3):bbae178. doi: 10.1093/bib/bbae178 (PMC11063651; doi:10.1093/bib/bbae178)
Supplement: embed_score_supp_bbae178 [file embed_score_supp_bbae178.pdf]

Scoring alignments by embedding vector similarity

– Supplementary material –

Sepehr Ashrafzadeh, G. Brian Golding, Silvana Ilie, Lucian Ilie*

1 Semi-global alignment tests

Supplementary Table 1: Semi-global alignments: ProtT5-score vs BLOSUM45 matrix, average distances for all five distances and all testing MSAs. Best results are shown in boldface. Wilcoxon test P-values higher than .01 are shown in red.

MSA					d_cc			d_d			d_pos			d_seq			d_ssp		
**Conserved domain**	**Source**	**Proteins**	**Length**	**Samples**	**ProtT5**	**BLOSUM45**	P-value	**ProtT5**	**BLOSUM45**	P-value	**ProtT5**	**BLOSUM45**	P-value	**ProtT5**	**BLOSUM45**	P-value	**ProtT5**	**BLOSUM45**	P-value
Bbox2_MID2_C-I	cd19823	7	40	21	0.000000	0.000000	-	0.000000	0.000000	-	0.000000	0.000000	-	0.000000	0.000000	-	0.000000	0.000000	-
Bbox2_TRIM42_C-III	cd19782	8	40	28	**0.003005**	0.007226	1.23E-01	0.000174	**0.001647**	1.17E-02	**0.016727**	0.045626	1.15E-02	**0.016727**	0.045626	1.15E-02	**0.020341**	0.030863	3.98E-01
Bbox2_MID	cd19758	9	40	36	**0.001193**	0.001380	7.20E-02	**0.000361**	0.000577	8.98E-03	**0.033052**	0.040788	3.79E-02	**0.033052**	0.040788	3.79E-02	0.044817	**0.036704**	6.36E-01
Bbox2_MID1_C-I	cd19822	8	47	28	0.000000	0.000000	-	0.000000	0.000000	-	0.000000	0.000000	-	0.000000	0.000000	-	0.000000	0.000000	-
Bbox_SF	cd00021	5	48	10	**0.023063**	0.048396	1.10E-01	**0.011057**	0.017582	6.78E-01	**0.236113**	0.334374	1.39E-01	**0.234950**	0.319753	1.73E-01	**0.241883**	0.324529	3.74E-01
DEFL_defensin-like	cd21806	77	51	1000	0.009939	**0.008986**	1.25E-12	**0.002986**	0.003824	1.19E-02	0.117725	**0.110485**	4.96E-05	0.102986	**0.096491**	2.14E-05	0.096388	**0.091258**	1.87E-05
Bbox2_TRIM37_C-VIII	cd19779	24	52	276	0.004326	**0.003388**	2.39E-05	0.001326	**0.001015**	1.92E-07	0.050800	**0.042909**	4.87E-03	0.044965	**0.035128**	1.36E-04	0.037020	**0.022129**	3.22E-13
Bbox2_TRIM9-like	cd19764	16	53	120	**0.006689**	0.011801	1.48E-02	**0.001876**	0.003876	6.02E-05	**0.066398**	0.099693	2.24E-06	**0.052880**	0.088069	1.27E-06	**0.049004**	0.078140	4.13E-03
CBD_like	cd12204	40	61	780	**0.010792**	0.020629	5.22E-67	**0.003655**	0.010030	9.30E-85	**0.151779**	0.231483	2.31E-82	**0.130193**	0.215568	4.12E-85	**0.157337**	0.220178	8.32E-47
Bbox2	cd19756	126	65	1000	**0.011706**	0.024913	1.60E-38	**0.003051**	0.006705	2.74E-34	**0.136067**	0.191049	7.99E-39	**0.128910**	0.184324	3.03E-39	**0.145214**	0.194423	3.62E-14
ChtBD1	cd00035	31	67	465	**0.012697**	0.022125	1.97E-23	**0.003355**	0.008155	7.09E-25	**0.160919**	0.219994	4.96E-23	**0.131405**	0.189655	1.49E-24	**0.128813**	0.187099	1.28E-19
Bbox1_CYLD	cd19816	26	68	325	**0.013685**	0.028400	1.85E-25	**0.004505**	0.007261	3.06E-09	**0.155255**	0.221111	1.36E-21	**0.146511**	0.212084	5.98E-21	**0.165157**	0.237162	2.90E-16
KAZAL_FS	cd00104	272	74	1000	**0.013331**	0.039055	8.09E-105	**0.003343**	0.013907	1.16E-100	**0.147045**	0.269885	1.12E-94	**0.135424**	0.257681	2.52E-94	**0.156723**	0.285979	1.92E-78
bHLH_SF	cd00083	78	75	1000	**0.007170**	0.028921	6.20E-83	**0.002888**	0.013312	2.14E-78	**0.099868**	0.217748	2.11E-81	**0.075181**	0.202779	1.58E-84	**0.073319**	0.224447	1.66E-82
CD_CSD	cd00024	521	98	1000	**0.009185**	0.023345	1.55E-79	**0.001518**	0.006105	2.92E-106	**0.107713**	0.197951	9.35E-93	**0.097490**	0.190341	2.30E-95	**0.100016**	0.218003	7.51E-93
C1	cd00029	280	99	1000	**0.013260**	0.022128	1.06E-58	**0.003753**	0.008317	4.86E-49	**0.161523**	0.221800	6.04E-48	**0.152520**	0.211229	1.67E-44	**0.169520**	0.237880	2.50E-39
TrHb	cd14756	7	130	21	**0.004535**	0.041555	8.86E-05	**0.001002**	0.007677	1.40E-04	**0.094067**	0.345955	8.86E-05	**0.083484**	0.338490	8.84E-05	**0.092226**	0.403291	8.86E-05
Hb	cd14765	14	138	91	**0.001490**	0.002487	5.10E-06	**0.000204**	0.000687	9.39E-09	**0.044115**	0.072793	4.59E-08	**0.034136**	0.066287	5.17E-10	**0.041652**	0.079085	5.05E-09
SH2_STAT5	cd10376	4	140	6	**0.001355**	0.002005	4.58E-01	**0.000121**	0.000146	4.58E-01	0.020036	**0.017497**	4.58E-01	0.018824	**0.016285**	4.58E-01	0.015888	**0.011003**	4.58E-01
Hb-beta-like	cd08925	26	140	325	**0.001183**	0.002039	1.98E-10	**0.000172**	0.000567	5.48E-16	**0.022683**	0.038984	5.58E-15	**0.020458**	0.036112	8.60E-14	**0.019597**	0.041014	2.34E-13
Hb-alpha-like	cd08927	38	142	703	**0.000628**	0.000770	3.41E-02	**0.000042**	0.000196	2.64E-07	**0.014554**	0.020563	9.25E-05	**0.014183**	0.020324	6.63E-05	**0.014483**	0.024913	4.22E-06
SH2_STAT5a	cd10421	9	145	36	**0.001055**	0.006663	4.57E-02	**0.000129**	0.000639	4.53E-02	**0.014668**	0.032758	4.53E-02	**0.011324**	0.030027	4.53E-02	**0.007680**	0.034564	4.53E-02
Mb	cd08926	8	149	28	0.000681	**0.000673**	8.33E-01	**0.000049**	0.000086	4.35E-04	**0.014150**	0.021647	3.82E-04	**0.013665**	0.021647	3.83E-04	**0.010430**	0.018105	3.44E-02
GS_GGDEF_2	cd14759	25	152	300	**0.001836**	0.012038	1.71E-45	**0.000270**	0.003370	5.51E-44	**0.027214**	0.166451	8.01E-48	**0.026258**	0.165448	1.16E-47	**0.034206**	0.209150	6.81E-42
Globin-like	cd01067	16	161	120	**0.032387**	0.160539	6.33E-21	**0.006550**	0.118599	5.43E-21	**0.370198**	0.806900	6.02E-21	**0.349277**	0.775463	5.30E-21	**0.410540**	0.823240	4.91E-21
FHb-globin	cd08922	46	162	1000	**0.001193**	0.002227	2.08E-46	**0.000136**	0.000582	2.85E-87	**0.024016**	0.042325	1.22E-78	**0.018197**	0.037830	1.32E-81	**0.017221**	0.037842	1.43E-70
PFM_HFR-2-like	cd20216	89	174	1000	**0.006854**	0.009347	1.47E-13	**0.001558**	0.002095	6.77E-26	**0.104309**	0.139559	1.15E-43	**0.103328**	0.138197	4.30E-42	**0.145610**	0.188414	9.28E-35
SH2_STAT_family	cd09919	66	206	1000	**0.020585**	0.037606	1.29E-95	**0.006556**	0.014647	1.99E-72	**0.211645**	0.305874	1.63E-109	**0.187197**	0.283813	2.68E-108	**0.216368**	0.316673	6.91E-96
PBP-like	cd08919	30	213	435	**0.012749**	0.038775	3.66E-62	**0.003603**	0.016699	2.28E-53	**0.198316**	0.353067	5.24E-64	**0.187027**	0.341131	5.79E-64	**0.229801**	0.398219	5.24E-60
SH2	cd00173	351	214	1000	**0.034202**	0.069256	1.18E-121	**0.009049**	0.028928	4.44E-113	**0.302374**	0.478800	4.13E-137	**0.278429**	0.457182	1.57E-136	**0.330741**	0.516657	1.39E-120
Globin_sensor	cd01068	192	223	1000	**0.004510**	0.023603	2.72E-149	**0.000599**	0.007730	5.95E-155	**0.067915**	0.257758	9.03E-156	**0.053022**	0.247491	1.20E-155	**0.062830**	0.301256	2.41E-152
PFM_monalysin-like	cd17904	30	229	435	**0.013733**	0.022054	1.96E-28	**0.004753**	0.006962	6.78E-13	**0.269102**	0.324128	1.16E-26	**0.266582**	0.321456	1.87E-26	**0.352371**	0.402670	1.49E-20
Mb-like	cd01040	383	239	1000	**0.008745**	0.049610	1.20E-159	**0.002168**	0.021932	4.83E-156	**0.168681**	0.454611	6.32E-160	**0.143730**	0.441055	3.34E-160	**0.167673**	0.506332	1.74E-159
FYVE_like_SF	cd00065	391	266	1000	**0.011427**	0.044924	1.58E-137	**0.004673**	0.023930	2.82E-134	**0.170099**	0.316531	5.81E-139	**0.103658**	0.253519	4.36E-144	**0.119199**	0.283590	7.80E-130
PFM_aerolysin_family	cd10140	64	270	1000	**0.045966**	0.077194	4.77E-152	**0.010275**	0.031625	9.34E-138	**0.640202**	0.759972	5.89E-118	**0.603952**	0.724957	5.51E-117	**0.715984**	0.810414	4.53E-99
7tmA_photoreceptors_insect	cd15079	34	301	561	**0.001341**	0.002463	3.10E-69	**0.000210**	0.001028	3.34E-75	**0.047872**	0.084988	5.98E-75	**0.045653**	0.083243	2.15E-75	**0.063282**	0.117193	7.43E-70
7tmA_Melanopsin-like	cd15083	11	314	55	**0.000879**	0.003872	2.15E-10	**0.000131**	0.001837	1.92E-10	**0.043049**	0.116844	2.15E-10	**0.034671**	0.110080	1.92E-10	**0.038653**	0.144452	2.40E-10
ClyA_AhlB-like	cd22652	38	354	703	**0.001717**	0.003703	4.19E-49	**0.000165**	0.000839	1.16E-81	**0.029657**	0.071668	1.55E-84	**0.028478**	0.070466	1.13E-83	**0.037583**	0.094774	3.23E-64
7tmA_Opsins_type2_animals	cd14969	70	400	1000	**0.002007**	0.008308	6.77E-163	**0.000578**	0.003734	1.15E-154	**0.070029**	0.232582	6.03E-164	**0.057368**	0.222365	4.05E-164	**0.067984**	0.287533	2.95E-163
7tm_GPCRs	cd14964	17	420	136	**0.022676**	0.098840	4.60E-24	**0.005327**	0.071275	5.61E-24	**0.533216**	0.891019	6.27E-24	**0.480151**	0.849745	5.74E-24	**0.609748**	0.912477	8.17E-24
7tmA_Anaphylatoxin_R-like	cd14974	17	429	136	**0.003931**	0.010849	1.56E-23	**0.001186**	0.003688	3.79E-22	**0.109928**	0.214792	6.71E-24	**0.080396**	0.188687	6.71E-24	**0.102004**	0.247353	7.51E-24
7tmA_Opioid_R-like	cd14970	15	458	105	**0.002042**	0.007125	7.78E-19	**0.000973**	0.003406	2.67E-17	**0.117470**	0.193901	3.50E-18	**0.047128**	0.130043	1.25E-18	**0.048755**	0.167504	8.78E-19
ClyA-like	cd21116	117	519	1000	**0.023298**	0.061872	1.25E-155	**0.013386**	0.037126	5.22E-145	**0.428380**	0.670217	1.10E-157	**0.379601**	0.647586	1.35E-158	**0.464798**	0.713613	4.64E-154
FGGY_RBK_like	cd07768	5	537	10	**0.005103**	0.008179	1.95E-03	**0.001284**	0.003311	1.95E-02	**0.227322**	0.315757	1.95E-03	**0.206354**	0.303679	1.95E-03	**0.282124**	0.381714	1.95E-03
FGGY	cd00366	30	588	435	**0.004487**	0.010982	2.14E-71	**0.002159**	0.004847	3.36E-45	**0.188844**	0.353850	7.58E-72	**0.178586**	0.346967	7.63E-72	**0.239448**	0.431288	9.42E-72
FGGY_YpCarbK_like	cd07782	34	690	561	**0.001643**	0.005296	5.73E-78	**0.000228**	0.001483	4.47E-82	**0.063658**	0.107713	3.03E-89	**0.051289**	0.097955	2.07E-89	**0.063410**	0.120201	5.06E-87
7tm_classA_rhodopsin-like	cd00637	404	808	1000	**0.005781**	0.026139	5.06E-165	**0.001896**	0.012196	1.28E-161	**0.184417**	0.452805	5.22E-165	**0.149100**	0.429213	5.23E-165	**0.193769**	0.525537	5.76E-165
NBD_sugar-kinase_HSP70_actin	cd00012	124	1154	1000	**0.034132**	0.116192	2.96E-161	**0.017823**	0.098320	1.18E-155	**0.662081**	0.888308	6.30E-164	**0.568667**	0.814094	1.52E-163	**0.689824**	0.901220	9.24E-164
7tmA_amine_R-like	cd14967	77	1227	1000	**0.004941**	0.021477	3.65E-164	**0.002561**	0.015229	2.10E-155	**0.191743**	0.355967	5.38E-165	**0.107736**	0.283244	5.38E-165	**0.140371**	0.357374	3.73E-165

*Corresponding author: ilie@uwo.ca

Supplementary Table 2: Semi-global alignments: ProtT5-score vs BLOSUM45 matrix, better cases.

MSA					d_cc			d_d			d_pos			d_seq			d_ssp		
**Conserved domain**	**Source**	**Proteins**	**Length**	**Samples**	**ProtT5**	**BLOSUM45**	Equal	**ProtT5**	**BLOSUM45**	Equal	**ProtT5**	**BLOSUM45**	Equal	**ProtT5**	**BLOSUM45**	Equal	**ProtT5**	**BLOSUM45**	Equal
Bbox2_MID2_C-I	cd19823	7	40	21	0	0	21	0	0	21	0	0	21	0	0	21	0	0	21
Bbox2_TRIM42_C-III	cd19782	8	40	28	**6**	2	20	**8**	0	20	**8**	0	20	**8**	0	20	**5**	2	21
Bbox2_MID	cd19758	9	40	36	**11**	3	22	**11**	1	24	**10**	2	24	**10**	2	24	**9**	5	22
Bbox2_MID1_C-I	cd19822	8	47	28	0	0	28	0	0	28	0	0	28	0	0	28	0	0	28
Bbox_SF	cd00021	5	48	10	**6**	3	1	**5**	4	1	**6**	3	1	**6**	3	1	**6**	3	1
DEFL_defensin-like	cd21806	77	51	1000	244	**454**	302	264	**415**	321	246	**388**	366	243	**400**	357	240	**411**	349
Bbox2_TRIM37_C-VIII	cd19779	24	52	276	58	**77**	141	45	**83**	148	45	**62**	169	46	**76**	154	9	**80**	187
Bbox2_TRIM9-like	cd19764	16	53	120	**60**	25	35	**64**	17	39	**67**	15	38	**67**	17	36	**43**	17	60
CBD_like	cd12204	40	61	780	**552**	124	104	**563**	102	115	**564**	84	132	**571**	81	128	**464**	146	170
Bbox2	cd19756	126	65	1000	**582**	285	133	**555**	267	178	**550**	213	237	**554**	223	223	**476**	308	216
ChtBD1	cd00035	31	67	465	**274**	119	72	**265**	121	79	**245**	115	105	**249**	115	101	**241**	130	94
Bbox1_CYLD	cd19816	26	68	325	**217**	68	40	**178**	98	49	**195**	67	63	**198**	69	58	**186**	80	59
KAZAL_FS	cd00104	272	74	1000	**685**	142	173	**662**	154	184	**660**	138	202	**664**	142	194	**621**	194	185
bHLH_SF	cd00083	78	75	1000	**607**	166	227	**588**	165	247	**601**	146	253	**601**	144	255	**599**	149	252
CD_CSD	cd00024	521	98	1000	**706**	193	101	**726**	148	126	**695**	155	150	**702**	159	139	**704**	159	137
C1	cd00029	280	99	1000	**626**	267	107	**591**	282	127	**595**	263	142	**589**	274	137	**579**	289	132
TrHb	cd14756	7	130	21	**20**	0	1	**19**	1	1	**20**	0	1	**20**	0	1	**20**	0	1
Hb	cd14765	14	138	91	**51**	16	24	**52**	15	24	**51**	16	24	**51**	13	27	**51**	13	27
SH2_STAT5	cd10376	4	140	6	**2**	**2**	2	**2**	**2**	2	**2**	**2**	2	**2**	2	2	**2**	**2**	2
Hb-beta-like	cd08925	26	140	325	**133**	46	146	**131**	46	148	**134**	41	150	**131**	45	149	**121**	40	164
Hb-alpha-like	cd08927	38	142	703	121	**143**	439	**132**	129	442	128	**131**	444	129	**131**	443	**132**	129	442
SH2_STAT5a	cd10421	9	145	36	**8**	6	22	**8**	6	22	**8**	6	22	**8**	6	22	**8**	6	22
Mb	cd08926	8	149	28	**13**	9	6	**16**	0	12	**16**	4	8	**16**	4	8	**12**	6	10
GS_GGDEF_2	cd14759	25	152	300	**276**	8	16	**274**	10	16	**279**	5	16	**279**	5	16	**244**	8	48
Globin-like	cd01067	16	161	120	**116**	2	2	**116**	2	2	**116**	2	2	**116**	2	2	**116**	2	2
FHb-globin	cd08922	46	162	1000	**573**	185	242	**581**	124	295	**577**	124	299	**581**	123	296	**551**	129	320
PFM_HFR-2-like	cd20216	89	174	1000	**476**	341	183	**515**	287	198	**539**	244	217	**538**	254	208	**523**	279	198
SH2_STAT_family	cd09919	66	206	1000	**734**	197	69	**669**	260	71	**748**	162	90	**747**	169	84	**717**	206	77
PBP-like	cd08919	30	213	435	**378**	40	17	**363**	54	18	**376**	37	22	**378**	36	21	**363**	49	23
SH2	cd00173	351	214	1000	**860**	139	1	**856**	143	1	**871**	99	30	**877**	106	17	**836**	151	13
Globin_sensor	cd01068	192	223	1000	**917**	49	34	**931**	29	40	**930**	26	44	**930**	27	43	**908**	37	55
PFM_monalysin-like	cd17904	30	229	435	**311**	112	12	**289**	136	10	**309**	102	24	**312**	99	24	**293**	124	18
Mb-like	cd01040	383	239	1000	**956**	34	10	**948**	42	10	**952**	32	16	**950**	35	15	**948**	40	12
FYVE_like_SF	cd00065	391	266	1000	**871**	91	38	**873**	76	51	**868**	63	69	**880**	61	59	**843**	89	68
PFM_aerolysin_family	cd10140	64	270	1000	**925**	72	3	**894**	103	3	**863**	129	8	**861**	131	8	**832**	162	6
7tmA_photoreceptors_insect	cd15079	34	301	561	**462**	71	28	**473**	58	30	**466**	58	37	**467**	58	36	**458**	64	39
7tmA_Melanopsin-like	cd15083	11	314	55	**52**	2	1	**52**	2	1	**52**	2	1	**52**	2	1	**52**	2	1
ClyA_AhlB-like	cd22652	38	354	703	**527**	87	89	**572**	42	89	**568**	42	93	**565**	48	90	**431**	50	222
7tmA_Opsins_type2_animals	cd14969	70	400	1000	**984**	10	6	**960**	34	6	**986**	7	7	**989**	5	6	**979**	12	9
7tm_GPCRs	cd14964	17	420	136	**136**	0	0	**134**	2	0	**134**	2	0	**134**	2	0	**134**	2	0
7tmA_Anaphylatoxin_R-like	cd14974	17	429	136	**133**	2	1	**131**	4	1	**135**	0	1	**135**	0	1	**134**	1	1
7tmA_Opioid_R-like	cd14970	15	458	105	**102**	3	0	**99**	6	0	**99**	5	1	**103**	0	2	**103**	1	1
ClyA-like	cd21116	117	519	1000	**942**	41	17	**909**	74	17	**934**	44	22	**942**	39	19	**916**	64	20
FGGY_RBK_like	cd07768	5	537	10	**10**	0	0	**9**	1	0	**10**	0	0	**10**	0	0	**10**	0	0
FGGY	cd00366	30	588	435	**418**	10	7	**348**	80	7	**426**	2	7	**426**	2	7	**425**	3	7
FGGY_YpCarbK_like	cd07782	34	690	561	**503**	53	5	**509**	48	4	**528**	24	9	**528**	24	9	**517**	32	12
7tm_classA_rhodopsin-like	cd00637	404	808	1000	**996**	3	1	**978**	21	1	**997**	2	1	**997**	2	1	**995**	4	1
NBD_sugar-kinase_HSP70_actin	cd00012	124	1154	1000	**968**	26	6	**928**	66	6	**983**	11	6	**975**	19	6	**980**	14	6
7tmA_amine_R-like	cd14967	77	1227	1000	**987**	12	1	**964**	36	0	**995**	4	1	**995**	4	1	**995**	5	0
**Total**					**19,595**	3,740	2,956	**19,260**	3,796	3,235	**19,587**	3,079	3,625	**19,632**	3,159	3,500	**18,831**	3,699	3,761
**Total %**					**0.75**	0.14	0.11	**0.73**	0.14	0.12	**0.75**	0.12	0.14	**0.75**	0.12	0.13	**0.72**	0.14	0.14
**Total for length over 300**					**7,220**	320	162	**7,066**	474	162	**7,313**	203	186	**7,318**	205	179	**7,129**	254	319
**Total % for length over 300**					**0.94**	0.04	0.02	**0.92**	0.06	0.02	**0.95**	0.03	0.02	**0.95**	0.03	0.02	**0.93**	0.03	0.04

2 Best *E*-score

Supplementary Table 3: Semi-global alignments: average distance for all E-scores and all five distances for six selected MSAs. Best results are shown in boldface.

MSA					d_cc					
Conserved domain	Source	Proteins	Length	Samples	ProtT5	ProtBert	ESM1b	ESM2	ProtAlbert	XLNet
Hb-alpha-like	cd08927	38	142	300	0.000678	**0.000510**	0.000628	0.000555	0.000635	-
Globin-like	cd01067	16	161	120	**0.032387**	0.049760	0.061074	0.035322	0.049610	0.06821
7tmA_photoreceptors_insect	cd15079	34	301	300	0.001399	0.001319	0.001241	**0.000967**	0.001390	0.00211
7tm_GPCRs	cd14964	17	420	136	**0.022676**	0.038388	0.031416	0.027154	0.033099	0.03873
FGGY_YpCarbK_like	cd07782	34	690	300	**0.001603**	0.001793	0.002231	0.001730	0.002565	-
NBD_sugar-kinase_HSP70_actin	cd00012	124	1154	300	**0.033371**	0.050246	0.046352	0.041707	0.049278	-

MSA					d_d						d_pos					
Conserved domain	Source	Proteins	Length	Samples	ProtT5	ProtBert	ESM1b	ESM2	ProtAlbert	XLNet	ProtT5	ProtBert	ESM1b	ESM2	ProtAlbert	XLNet
Hb-alpha-like	cd08927	38	142	300	0.000044	0.000037	0.000045	**0.000037**	0.000048	-	0.015327	0.012939	0.013942	**0.012800**	0.015381	-
Globin-like	cd01067	16	161	120	**0.006550**	0.015153	0.017836	0.008764	0.011445	0.02027	**0.370198**	0.551089	0.613001	0.401669	0.544223	0.68865
7tmA_photoreceptors_insect	cd15079	34	301	300	0.000228	0.000186	0.000157	**0.000145**	0.000184	0.00038	0.049456	0.046965	0.045116	**0.035749**	0.045387	0.07241
7tm_GPCRs	cd14964	17	420	136	**0.005327**	0.018973	0.012929	0.011427	0.009080	0.01586	**0.533216**	0.686566	0.637672	0.556423	0.668073	0.69320
FGGY_YpCarbK_like	cd07782	34	690	300	**0.000224**	0.000267	0.000438	0.000240	0.000385	-	0.063175	0.068498	0.078059	**0.061100**	0.071485	-
NBD_sugar-kinase_HSP70_actin	cd00012	124	1154	300	**0.018078**	0.037083	0.035718	0.030538	0.034399	-	**0.664004**	0.795209	0.750176	0.709844	0.785006	-

MSA					d_seq						d_ssp					
Conserved domain	Source	Proteins	Length	Samples	ProtT5	ProtBert	ESM1b	ESM2	ProtAlbert	XLNet	ProtT5	ProtBert	ESM1b	ESM2	ProtAlbert	XLNet
Hb-alpha-like	cd08927	38	142	300	0.014910	0.012677	0.013799	**0.012467**	0.015167	-	0.015378	0.010555	0.014715	**0.011833**	0.016289	-
Globin-like	cd01067	16	161	120	**0.349277**	0.544652	0.604901	0.388417	0.530987	0.68365	**0.410540**	0.617252	0.674917	0.452781	0.605535	0.75062
7tmA_photoreceptors_insect	cd15079	34	301	300	0.047164	0.044023	0.042024	**0.032658**	0.043924	0.06998	0.065482	0.063650	0.063435	**0.046808**	0.062535	0.10647
7tm_GPCRs	cd14964	17	420	136	**0.480151**	0.664946	0.601501	0.523269	0.628612	0.66842	**0.609748**	0.753075	0.712301	0.629215	0.738981	0.76306
FGGY_YpCarbK_like	cd07782	34	690	300	0.050921	0.058269	0.067059	**0.050394**	0.060601	-	0.062732	0.068537	0.093033	**0.060126**	0.072615	-
NBD_sugar-kinase_HSP70_actin	cd00012	124	1154	300	**0.565443**	0.748106	0.698232	0.656375	0.732283	-	**0.688703**	0.823625	0.781364	0.733834	0.814248	-

Supplementary Table 4: Semi-global alignments: ProtT5-score vs ESM2-score for six selected MSAs. Best results are shown in boldface. Wilcoxon test P-values higher than .01 are shown in red.

MSA					d_cc			d_d			d_pos			d_seq			d_ssp		
Conserved domain	Source	Proteins	Length	Samples	ESM2	ProtT5	P-value	ESM2	ProtT5	P-value	ESM2	ProtT5	P-value	ESM2	ProtT5	P-value	ESM2	ProtT5	P-value
Hb-alpha-like	cd08927	38	142	300	**0.000555**	0.000678	9.32E-07	**0.000037**	0.000044	3.39E-05	**0.012800**	0.015327	1.65E-07	**0.012467**	0.014910	4.98E-07	**0.011833**	0.015378	4.29E-06
Globin-like	cd01067	16	161	120	0.035322	**0.032387**	1.51E-02	0.008764	**0.006550**	2.26E-11	0.401669	**0.370198**	5.40E-03	0.388417	**0.349277**	1.26E-03	0.452781	**0.410540**	1.55E-03
7tmA_photoreceptors_insect	cd15079	34	301	300	**0.000967**	0.001399	1.73E-29	**0.000145**	0.000228	9.58E-38	**0.035749**	0.049456	1.74E-39	**0.032658**	0.047164	8.10E-40	**0.046808**	0.065482	2.07E-38
7tm_GPCRs	cd14964	17	420	136	0.027154	**0.022676**	6.58E-04	0.011427	**0.005327**	3.87E-10	0.556423	**0.533216**	3.83E-02	0.523269	**0.480151**	2.49E-04	0.629215	**0.609748**	1.04E-01
FGGY_YpCarbK_like	cd07782	34	690	300	0.001730	**0.001603**	3.31E-03	0.000240	**0.000224**	1.66E-03	**0.061100**	0.063175	1.07E-03	**0.050394**	0.050921	4.10E-02	**0.060126**	0.062732	1.66E-08
NBD_sugar-kinase_HSP70_actin	cd00012	124	1154	300	0.041707	**0.033371**	1.90E-18	0.030538	**0.018078**	1.85E-32	0.709844	**0.664004**	1.12E-16	0.656375	**0.565443**	1.34E-30	0.733834	**0.688703**	8.20E-15

3 Best BLOSUM matrix

Supplementary Table 5: Semi-global alignments: average distance for all BLOSUM matrices and all five distances for six selected MSAs. Best results are shown in boldface.

MSA					d_cc				
Conserved domain	Source	Proteins	Length	Samples	BLOSUM45	BLOSUM50	BLOSUM62	BLOSUM80	BLOSUM90
Hb-alpha-like	cd08927	38	142	300	0.000975	0.001074	**0.000870**	0.001106	0.001191
Globin-like	cd01067	16	161	120	**0.160539**	0.163812	0.311554	0.384547	0.382977
7tmA_photoreceptors_insect	cd15079	34	301	300	0.002451	0.002687	**0.002305**	0.002681	0.002876
7tm_GPCRs	cd14964	17	420	136	**0.098840**	0.114038	0.291492	0.442796	0.444632
FGGY_YpCarbK_like	cd07782	34	690	300	0.004856	0.004930	**0.004560**	0.004953	0.005228
NBD_sugar-kinase_HSP70_actin	cd00012	124	1154	300	**0.120524**	0.124483	0.351562	0.436627	0.443355

MSA					d_d					d_pos				
Conserved domain	Source	Proteins	Length	Samples	BLOSUM45	BLOSUM50	BLOSUM62	BLOSUM80	BLOSUM90	BLOSUM45	BLOSUM50	BLOSUM62	BLOSUM80	BLOSUM90
Hb-alpha-like	cd08927	38	142	300	0.000228	0.000332	**0.000172**	0.000313	0.000557	0.022717	0.025696	**0.021142**	0.027009	0.029803
Globin-like	cd01067	16	161	120	**0.118599**	0.124467	0.292858	0.376061	0.373764	**0.806900**	0.816397	0.846399	0.876281	0.881988
7tmA_photoreceptors_insect	cd15079	34	301	300	0.001084	0.001500	**0.000857**	0.001484	0.002018	0.084005	0.090461	**0.079186**	0.089835	0.097210
7tm_GPCRs	cd14964	17	420	136	**0.071275**	0.092714	0.291051	0.441464	0.444240	**0.891019**	0.902100	0.931473	0.972709	0.976773
FGGY_YpCarbK_like	cd07782	34	690	300	0.001399	0.001774	**0.001235**	0.001788	0.002313	0.107904	0.111766	**0.101768**	0.111036	0.117510
NBD_sugar-kinase_HSP70_actin	cd00012	124	1154	300	**0.104434**	0.109325	0.351568	0.435391	0.443592	**0.903067**	0.904574	0.939824	0.954303	0.956641

MSA					d_seq					d_ssp				
Conserved domain	Source	Proteins	Length	Samples	BLOSUM45	BLOSUM50	BLOSUM62	BLOSUM80	BLOSUM90	BLOSUM45	BLOSUM50	BLOSUM62	BLOSUM80	BLOSUM90
Hb-alpha-like	cd08927	38	142	300	0.022455	0.025446	**0.020892**	0.026795	0.029518	0.027156	0.031297	**0.024384**	0.031611	0.033224
Globin-like	cd01067	16	161	120	**0.775463**	0.781519	0.786103	0.801414	0.806853	**0.823240**	0.830480	0.853761	0.879902	0.884695
7tmA_photoreceptors_insect	cd15079	34	301	300	0.082066	0.088500	**0.077444**	0.087955	0.095279	0.114693	0.121905	**0.107431**	0.119600	0.127150
7tm_GPCRs	cd14964	17	420	136	0.849745	0.856005	**0.848253**	0.867307	0.871364	**0.912477**	0.920913	0.942708	0.975132	0.978878
FGGY_YpCarbK_like	cd07782	34	690	300	0.097829	0.101641	**0.091791**	0.100644	0.106800	0.121254	0.124388	**0.112235**	0.122356	0.128248
NBD_sugar-kinase_HSP70_actin	cd00012	124	1154	300	0.825004	0.819877	0.761822	**0.747700**	0.748795	0.913651	**0.913441**	0.945692	0.957563	0.959222

Supplementary Table 6: Semi-global alignments: BLOSUM45 vs BLOSUM62 matrices for six selected MSAs. Best results are shown in boldface. Wilcoxon test P-values higher than .01 are shown in red.

MSA					d_cc			d_d			d_pos			d_seq			d_ssp		
Conserved domain	Source	Proteins	Length	Samples	BLOSUM45	BLOSUM62	P-value	BLOSUM45	BLOSUM62	P-value	BLOSUM45	BLOSUM62	P-value	BLOSUM45	BLOSUM62	P-value	BLOSUM45	BLOSUM62	P-value
Hb-alpha-like	cd08927	38	142	300	0.000975	**0.000870**	5.35E-01	0.000228	**0.000172**	3.72E-01	0.022717	**0.021142**	6.94E-01	0.022455	**0.020892**	6.87E-01	0.027156	**0.024384**	3.24E-01
Globin-like	cd01067	16	161	120	**0.160539**	0.311554	9.87E-16	**0.118599**	0.292858	2.21E-15	**0.806900**	0.846399	1.32E-06	**0.775463**	0.786103	9.58E-01	**0.823240**	0.853761	3.55E-06
7tmA_photoreceptors_insect	cd15079	34	301	300	0.002451	**0.002305**	2.30E-05	0.001084	**0.000857**	1.64E-05	0.084005	**0.079186**	5.50E-06	0.082066	**0.077444**	2.12E-05	0.114693	**0.107431**	1.82E-06
7tm_GPCRs	cd14964	17	420	136	**0.098840**	0.291492	8.75E-20	**0.071275**	0.291051	3.42E-19	**0.891019**	0.931473	1.82E-08	0.849745	**0.848253**	2.84E-02	**0.912477**	0.942708	5.50E-08
FGGY_YpCarbK_like	cd07782	34	690	300	0.004856	**0.004560**	1.25E-09	0.001399	**0.001235**	2.73E-06	0.107904	**0.101768**	2.21E-12	0.097829	**0.091791**	1.07E-11	0.121254	**0.112235**	4.35E-12
NBD_sugar-kinase_HSP70_actin	cd00012	124	1154	300	**0.120524**	0.351562	1.71E-46	**0.104434**	0.351568	2.38E-46	**0.903067**	0.939824	3.71E-24	0.825004	**0.761822**	4.53E-26	**0.913651**	0.945692	5.84E-24

4 Global alignment tests

Supplementary Table 7: Global alignments: ProtT5-score vs BLOSUM45 matrix, average distances for all five distances and all testing MSAs. Best results are shown in boldface. Wilcoxon test P-values higher than .01 are shown in red.

MSA					d_cc			d_d			d_pos			d_seq			d_ssp		
**Conserved domain**	**Source**	**Proteins**	**Length**	**Samples**	**ProtT5**	**BLOSUM45**	P-value	**ProtT5**	**BLOSUM45**	P-value	**ProtT5**	**BLOSUM45**	P-value	**ProtT5**	**BLOSUM45**	P-value	**ProtT5**	**BLOSUM45**	P-value
Bbox2_MID2_C-I	cd19823	7	40	21	0.000000	0.000000	-	0.000000	0.000000	-	0.000000	0.000000	-	0.000000	0.000000	-	0.000000	0.000000	-
Bbox2_TRIM42_C-III	cd19782	8	40	28	0.003005	**0.001694**	6.79E-02	0.000174	**0.000081**	6.79E-02	0.016727	**0.008590**	6.79E-02	0.016727	**0.008590**	6.79E-02	0.020341	**0.008104**	6.79E-02
Bbox2_MID	cd19758	9	40	36	0.001193	**0.001006**	4.31E-02	0.000361	**0.000289**	3.94E-02	0.033052	**0.026723**	3.94E-02	0.033052	**0.026723**	3.94E-02	0.044817	**0.035434**	3.94E-02
Bbox2_MID1_C-I	cd19822	8	47	28	0.000000	0.000000	-	0.000000	0.000000	-	0.000000	0.000000	-	0.000000	0.000000	-	0.000000	0.000000	-
Bbox_SF	cd00021	5	48	10	**0.023063**	0.031005	4.84E-01	**0.011057**	0.011504	8.89E-01	**0.236113**	0.246558	8.89E-01	0.234950	**0.231937**	8.66E-01	0.241883	**0.240388**	8.66E-01
DEFL_defensin-like	cd21806	77	51	300	0.010128	**0.008159**	6.34E-07	0.002951	**0.002898**	2.65E-02	0.119022	**0.111145**	1.72E-02	0.104142	**0.096748**	1.62E-02	0.097772	**0.097251**	1.80E-01
Bbox2_TRIM37_C-VIII	cd19779	24	52	276	0.004326	**0.003154**	6.05E-09	0.001326	**0.000833**	3.58E-14	0.050800	**0.034616**	1.25E-13	0.044965	**0.026834**	1.18E-14	0.037020	**0.021853**	3.59E-13
Bbox2_TRIM9-like	cd19764	16	53	120	0.006689	**0.004010**	6.92E-05	0.001876	**0.001368**	1.08E-02	0.066398	**0.054257**	4.93E-03	0.052880	**0.043590**	3.37E-02	0.049004	**0.043404**	6.68E-02
CBD_like	cd12204	40	61	300	**0.010804**	0.014363	6.95E-10	**0.003781**	0.005127	2.61E-08	**0.152429**	0.184270	1.11E-11	**0.130087**	0.166340	1.45E-13	**0.151414**	0.185038	2.02E-06
Bbox2	cd19756	126	65	300	**0.011511**	0.012688	9.71E-01	**0.002942**	0.003390	2.56E-01	0.134007	**0.130947**	3.43E-03	0.127192	**0.124068**	9.75E-03	0.143595	**0.140827**	8.86E-03
ChtBD1	cd00035	31	67	300	**0.012408**	0.014017	1.23E-02	**0.003179**	0.004553	2.66E-05	**0.157288**	0.176369	7.37E-04	**0.128726**	0.147971	2.05E-04	**0.124246**	0.149246	2.25E-04
Bbox1_CYLD	cd19816	26	68	325	**0.013647**	0.017508	1.81E-05	**0.004508**	0.005065	9.85E-01	**0.154754**	0.169441	2.77E-03	**0.146010**	0.160007	2.65E-03	**0.164416**	0.186957	4.62E-04
KAZAL_FS	cd00104	272	74	300	**0.013922**	0.028184	3.84E-22	**0.003314**	0.008707	1.79E-22	**0.145909**	0.227736	2.31E-20	**0.136510**	0.218210	5.27E-20	**0.158976**	0.250152	5.22E-17
bHLH_SF	cd00083	78	75	300	**0.007183**	0.014852	1.27E-18	**0.002838**	0.006743	1.51E-17	**0.102140**	0.163804	4.04E-16	**0.079882**	0.149265	3.48E-18	**0.079658**	0.171462	1.25E-18
CD_CSD	cd00024	521	98	300	**0.009396**	0.013120	4.39E-13	**0.001655**	0.003690	7.34E-25	**0.110263**	0.157445	2.75E-19	**0.100134**	0.149348	1.52E-20	**0.105011**	0.177089	4.61E-22
C1	cd00029	280	99	300	**0.013681**	0.018442	1.07E-10	**0.003766**	0.005289	1.61E-06	**0.165077**	0.204748	3.38E-09	**0.156099**	0.193662	5.46E-08	**0.174556**	0.226230	2.90E-08
TrHb	cd14756	7	130	21	**0.004520**	0.025456	1.32E-04	**0.000995**	0.005879	2.14E-04	**0.092407**	0.290605	1.32E-04	**0.081824**	0.283148	1.32E-04	**0.089073**	0.357702	1.32E-04
Hb	cd14765	14	138	91	**0.001490**	0.002370	8.92E-06	**0.000204**	0.000583	9.73E-09	**0.044115**	0.069436	4.91E-08	**0.034136**	0.062930	5.16E-10	**0.041652**	0.076554	5.03E-09
SH2_STAT5	cd10376	4	140	6	**0.001355**	0.002005	4.58E-01	**0.000121**	0.000146	4.58E-01	0.020036	**0.017497**	4.58E-01	0.018824	**0.016285**	4.58E-01	0.015888	**0.011003**	4.58E-01
Hb-beta-like	cd08925	26	140	300	**0.001107**	0.001862	4.23E-06	**0.000179**	0.000499	2.00E-09	**0.023368**	0.034923	7.42E-09	**0.021182**	0.032321	5.40E-08	**0.020466**	0.038795	4.80E-10
Hb-alpha-like	cd08927	38	142	300	**0.000678**	0.000937	5.06E-03	**0.000044**	0.000200	2.86E-04	**0.015327**	0.021191	6.51E-03	**0.014910**	0.020930	5.40E-03	**0.015378**	0.025827	9.99E-04
SH2_STAT5a	cd10421	9	145	36	**0.001055**	0.001406	1.16E-01	**0.000129**	0.000151	1.01E-01	**0.014668**	0.016317	7.81E-02	**0.011324**	0.013092	7.81E-02	**0.007680**	0.010716	7.81E-02
Mb	cd08926	8	149	28	0.000681	**0.000653**	3.79E-01	**0.000049**	0.000072	2.53E-02	**0.014150**	0.017551	1.95E-01	**0.013665**	0.017551	8.69E-02	**0.010430**	0.018042	2.59E-02
GS_GGDEF_2	cd14759	25	152	300	**0.001836**	0.007858	2.33E-28	**0.000270**	0.002425	1.16E-32	**0.027214**	0.122792	2.15E-35	**0.026258**	0.121768	4.57E-35	**0.034206**	0.170564	1.52E-34
Globin-like	cd01067	16	161	120	**0.031733**	0.064032	1.21E-17	**0.006543**	0.026334	1.26E-20	**0.368170**	0.659491	1.23E-20	**0.347358**	0.642125	7.01E-21	**0.408049**	0.710038	7.19E-21
FHb-globin	cd08922	46	162	300	**0.001234**	0.002119	3.83E-12	**0.000146**	0.000537	1.76E-25	**0.025158**	0.038901	6.24E-21	**0.019062**	0.034087	3.90E-22	**0.018162**	0.035502	3.70E-20
PFM_HFR-2-like	cd20216	89	174	300	0.006101	**0.005901**	7.67E-01	**0.001517**	0.001679	1.41E-04	**0.098214**	0.113686	2.92E-06	**0.097322**	0.112365	3.91E-06	**0.137490**	0.158859	5.70E-06
SH2_STAT_family	cd09919	66	206	300	**0.019460**	0.021276	1.34E-03	**0.005518**	0.006777	1.66E-03	**0.203658**	0.252028	2.34E-19	**0.178629**	0.224965	1.22E-16	**0.208088**	0.264876	7.82E-18
PBP-like	cd08919	30	213	300	**0.011902**	0.023623	3.30E-37	**0.003315**	0.007036	3.67E-25	**0.189161**	0.291599	3.08E-36	**0.176688**	0.280838	9.80E-36	**0.215297**	0.342865	1.09E-35
SH2	cd00173	351	214	300	**0.033438**	0.045111	7.33E-22	**0.009190**	0.013590	2.27E-20	**0.299843**	0.410207	1.17E-30	**0.275217**	0.388500	4.20E-30	**0.327982**	0.463043	2.24E-29
Globin_sensor	cd01068	192	223	300	**0.004459**	0.016223	1.49E-44	**0.000583**	0.004483	6.65E-47	**0.068155**	0.208243	3.84E-46	**0.051818**	0.197550	1.50E-46	**0.061683**	0.250642	5.03E-46
PFM_monalysin-like	cd17904	30	229	300	**0.013546**	0.015735	6.40E-07	**0.005046**	0.005114	3.71E-06	**0.263529**	0.286888	1.36E-07	**0.261302**	0.284632	2.12E-07	**0.346110**	0.372784	6.82E-07
Mb-like	cd01040	383	239	300	**0.008814**	0.025598	3.67E-45	**0.002241**	0.009006	5.63E-43	**0.166566**	0.359807	1.85E-46	**0.141470**	0.346156	7.23E-47	**0.163770**	0.416792	9.51E-47
FYVE_like_SF	cd00065	391	266	300	**0.012167**	0.031618	4.97E-41	**0.005300**	0.013507	1.80E-36	**0.181647**	0.292302	6.76E-39	**0.108226**	0.224827	5.07E-41	**0.123816**	0.259494	5.38E-39
PFM_aerolysin_family	cd10140	64	270	300	**0.045061**	0.056760	4.10E-26	**0.010532**	0.019177	3.29E-29	**0.642903**	0.721366	3.55E-21	**0.608259**	0.693639	3.82E-23	**0.719626**	0.787133	4.20E-18
7tmA_photoreceptors_insect	cd15079	34	301	300	**0.001399**	0.002368	1.55E-31	**0.000228**	0.001052	5.02E-34	**0.049456**	0.075499	7.19E-31	**0.047164**	0.073559	2.90E-31	**0.065482**	0.105396	1.89E-31
7tmA_Melanopsin-like	cd15083	11	314	55	**0.000879**	0.003824	8.74E-10	**0.000131**	0.001804	8.74E-10	**0.043049**	0.109751	1.36E-09	**0.034671**	0.102987	1.09E-09	**0.038653**	0.138049	1.52E-09
ClyA_AhlB-like	cd22652	38	354	300	**0.001996**	0.002498	6.75E-08	**0.000187**	0.000827	1.90E-20	**0.033045**	0.063414	2.11E-23	**0.031856**	0.061980	1.40E-22	**0.042178**	0.088833	6.11E-23
7tmA_Opsins_type2_animals	cd14969	70	400	300	**0.001943**	0.007413	1.31E-49	**0.000562**	0.003557	7.58E-48	**0.069111**	0.207764	2.95E-50	**0.056882**	0.197296	2.92E-50	**0.067174**	0.260493	3.13E-50
7tm_GPCRs	cd14964	17	420	136	**0.021977**	0.046764	1.22E-22	**0.005309**	0.022659	1.04E-23	**0.530298**	0.801367	5.48E-23	**0.477233**	0.766608	2.29E-23	**0.607210**	0.845613	1.24E-22
7tmA_Anaphylatoxin_R-like	cd14974	17	429	136	**0.003833**	0.008599	2.66E-22	**0.001186**	0.003628	1.11E-21	**0.109582**	0.198233	2.81E-23	**0.080050**	0.172128	3.84E-23	**0.101411**	0.229464	3.08E-23
7tmA_Opioid_R-like	cd14970	15	458	105	**0.002042**	0.006304	7.78E-19	**0.000973**	0.002897	2.67E-17	**0.117470**	0.191432	3.60E-18	**0.047128**	0.126863	1.25E-18	**0.048755**	0.165182	8.78E-19
ClyA-like	cd21116	117	519	300	**0.022690**	0.037783	2.74E-34	**0.013635**	0.024122	2.53E-30	**0.429975**	0.624160	1.20E-46	**0.376312**	0.589775	2.56E-47	**0.462390**	0.679178	4.11E-46
FGGY_RBK_like	cd07768	5	537	10	**0.005103**	0.007279	1.95E-03	**0.001284**	0.003284	1.95E-02	**0.227322**	0.311245	1.95E-03	**0.206354**	0.299167	1.95E-03	**0.282124**	0.378646	1.95E-03
FGGY	cd00366	30	588	300	**0.004575**	0.009960	1.05E-48	**0.002272**	0.004811	1.89E-34	**0.188870**	0.352219	2.74E-49	**0.178342**	0.344821	1.84E-49	**0.238063**	0.429620	2.43E-49
FGGY_YpCarbK_like	cd07782	34	690	300	**0.001603**	0.002904	3.06E-37	**0.000224**	0.001088	7.15E-45	**0.063175**	0.101153	7.85E-48	**0.050921**	0.090108	1.68E-47	**0.062732**	0.115266	3.31E-47
7tm_classA_rhodopsin-like	cd00637	404	808	300	**0.006051**	0.019284	6.46E-51	**0.002059**	0.007700	3.58E-49	**0.188327**	0.417532	6.79E-51	**0.154874**	0.393975	6.79E-51	**0.201403**	0.496238	7.00E-51
NBD_sugar-kinase_HSP70_actin	cd00012	124	1154	300	**0.032877**	0.052502	1.73E-44	**0.018053**	0.036214	3.38E-41	**0.663322**	0.838091	1.47E-50	**0.564686**	0.776516	1.29E-50	**0.687956**	0.860119	1.49E-50
7tmA_amine_R-like	cd14967	77	1227	300	**0.004752**	0.012643	6.34E-50	**0.002395**	0.008252	1.50E-46	**0.184162**	0.326660	1.22E-50	**0.106436**	0.252929	1.15E-50	**0.137242**	0.326527	8.22E-51

5 Matrices heatmaps

Supplementary Table 8: All five BLOSUM matrices and all twelve E-score matrices for the *NBD_sugar-kinase_HSP70_actin* MSA.

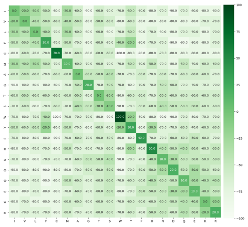
BLOSUM45

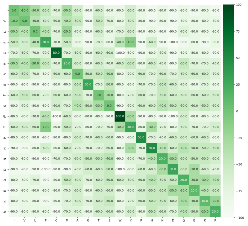
BLOSUM50

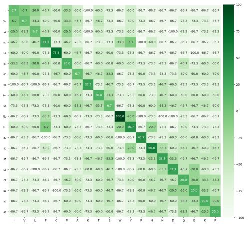
BLOSUM62

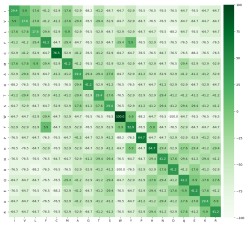
BLOSUM80

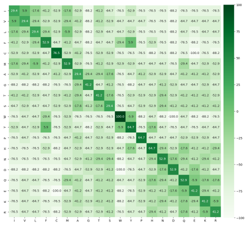
BLOSUM90

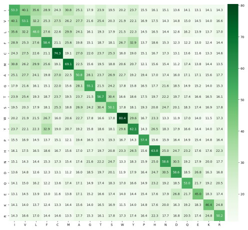
ProtT5 (A)

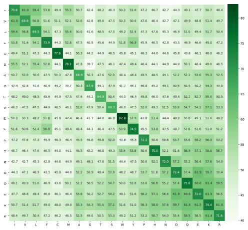
ESM2 (A)

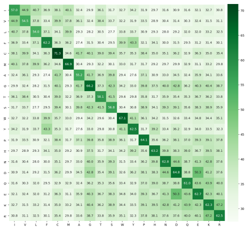
ProtBert (A)

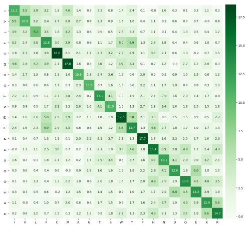
ProtT5 (U)

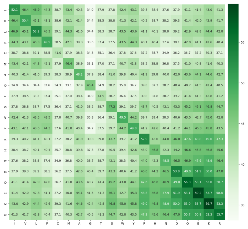
ESM2 (U)

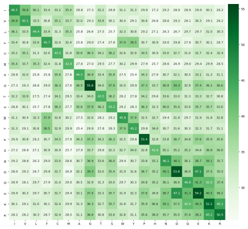
ProtBert (U)

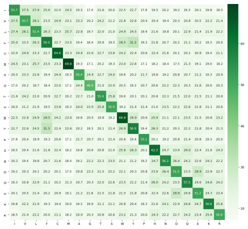
ProtAlbert (A)

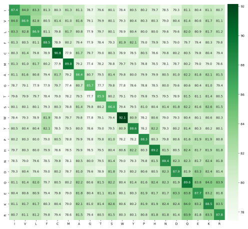
ESM-1b (A)

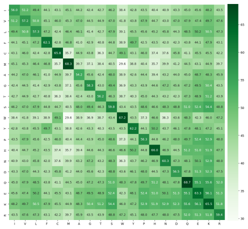
ProtXLNet (A)

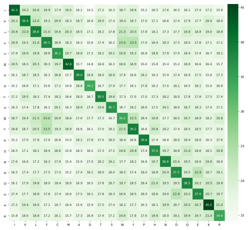
ProtAlbert (U)

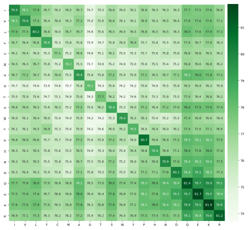
ESM-1b (U)

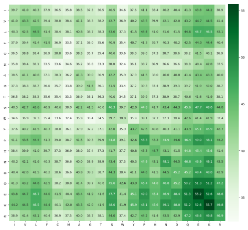
ProtXLNet (U)
